# Comparing Social Media Observations of Animals During a Solar Eclipse to Published Research

**DOI:** 10.3390/ani9020059

**Published:** 2019-02-14

**Authors:** Robert Ritson, Dustin H. Ranglack, Nate Bickford

**Affiliations:** Department of Biology, University of Nebraska at Kearney, Kearney, NE 68849, USA; ritsonrj@lopers.unk.edu (R.R.); ranglackdh@unk.edu (D.H.R.)

**Keywords:** eclipse, solar, animals, behavior

## Abstract

**Simple Summary:**

Solar eclipses offer a unique opportunity to evaluate the relative influence of unexpected darkness on behavior of some species of animals due to their sudden interference with local light levels and meteorology. The Great American Solar Eclipse of 21 August 2017 rekindled curiosity in animal behavior during an eclipse. What made this most recent eclipse especially unique was the fact that it occurred over a relatively populous region of the globe, with approximately 12 million people living in the path of totality, garnering a lot of publicity. This immense viewership created a unique opportunity to gather a large amount of observations simultaneously across the eclipse. A comparison of informal observations of animal behavior during solar eclipse from social media (i.e., March for Science Facebook discussion) to those conducted scientifically (published literature) can elucidate how well this topic is being covered. Describing which species and behaviors are covered in each source can reveal gaps in the literature which can emphasize areas for future research. Our understanding of animal behavior can benefit beyond the narrow scope of such studies by characterizing the complex variations in behavioral response which result from a solar eclipse.

**Abstract:**

A wide variety of environmental stimuli can influence the behavior of animals including temperature, weather, light, lunar and seasonal cycles, seismic activity, as well as other perturbations to their circadian rhythm. Solar eclipses offer a unique opportunity to evaluate the relative influence of unexpected darkness on behavior of animals due to their sudden interference with local light levels and meteorology. Though occasionally bizarre, modern studies have lent support to the idea that at least some individuals of certain species display altered behavior during these events. A comparison of informal observations of animal behavior during solar eclipse from social media (i.e., March for Science Facebook discussion) to those conducted scientifically (published literature) can elucidate how well this topic is being covered. Describing which species and behaviors are covered in each source can reveal gaps in the literature which can emphasize areas for future research. We enumerated a total of 685 observations of approximately 48 different types of animals reacting to the 2017 Great American Solar Eclipse from over 800 posts on the discussion. The animals most frequently reported on social media as reacting to the eclipse were invertebrates (40% of social media observations) and birds (35% of social media observations). A total of 26 published studies recorded 169 behavior observations of approximately 131 different animal species. The group with the highest number of observations in the literature were birds with 62 records (37% of literature observations). Most observations reported decreases in activity (38.7% of bird observations) followed by increases in vocalization (24.2% of bird observations). There were approximately 30 different species of invertebrate observed (24% of literature observations), most frequently reported of which were zooplankton (14.6% of invertebrate observations).

## 1. Introduction

A wide variety of environmental stimuli can influence the behavior of animals including temperature, weather, light, lunar and seasonal cycles, seismic activity, as well as other perturbations to their circadian rhythm [1]. The manner in which animals respond to these varying conditions is often related to their specific life history [2], including physiology and orientation–navigational behaviors [3]. Solar eclipses offer a unique opportunity to evaluate the relative influence of unexpected darkness on relatively prolonged behavior due to their sudden interference with local light levels and meteorology [4]. However, planning experimental research studies around these novel events is often difficult. Although they occur somewhere on Earth two to five times in a given calendar year, a given location will experience total darkness only once every 350 years for a maximum totality duration of seven minutes although typically around three minutes [5]. The mechanics responsible for these phenomena are well understood [6], but our comprehension of how or why animals react to such events is surprisingly limited. Despite the meager attention, studies of animal behavior during solar eclipses have been published for a variety species.

The first recorded observation of an animal responding to a solar eclipse, to our knowledge, was made in mid-1500 noting “birds falling out of the sky and ceasing to sing” [7]. Though occasionally bizarre, modern studies have lent support to the idea that at least some individuals of certain species display altered behavior during these events. In addition to further observations on birds [8,9], the list of investigated species includes insects [10,11,12,13,14], aquatic invertebrates [15,16,17,18,19], primates [20,21,22,23], fish [24,25], rodents [26,27], bats [28,29], and lizards [30,31], as well as larger mammals like blue bull antelope [32] and dairy cattle [33]. However, conclusions vary by study and species. For example, the studies on bats reach similar conclusions which suggest no change in behavior [28,29] but observations of chimpanzees differ [20,22]. Studies of animal behavior during solar eclipses tend to be brief in length compared to typical studies in this field and usually rely on relatively simple observational protocols to document activity. However, recent studies have incorporated more sophisticated methods to explore this topic including radar documenting the activity of flying animals [34] and acoustic monitoring of calling activities [35].

The Great American Solar Eclipse of August 21, 2017 rekindled curiosity in animal behavior during an eclipse [34,35]. What made this most recent eclipse especially unique was the fact that it occurred over a relatively populous region of the globe, with approximately 12 million people living in the path of totality—garnering a lot of publicity [36]—many of whom own domestic animals. This immense viewership created a unique opportunity to gather a large amount of animal observations simultaneously across the eclipse. Although more efficient and scientifically robust methods exist for formally procuring observations (i.e., surveys, mobile applications, etc.), informal notes are often overlooked. Since this topic has received little formal attention, informal accounts can offer useful insight for what future work should address. By reviewing social media posts concerning animal behavior during the 2017 solar eclipse, species or behaviors not otherwise considered by scientists may be procured. We chose to explore a Facebook discussion post on the March for Science page which solicited observations of animal behaviors during the eclipse [37]. This social media platform also lends itself to detailed statements than others which have fixed character limits (i.e., Twitter). It was inferred that members of this group would not be restricted to a single geographic area, thus increasing the region observed. Although contributed posts were usually nonspecific, these anecdotal observations have the potential to expose behaviors or species not currently addressed in the literature. These may indicate which species should be focused on for future studies of animal behavior during solar eclipses and how such investigations may be improved.

The objective of this investigation was to compare informal observations of animal behavior during solar eclipse from social media (i.e., March for Science Facebook discussion) [37] to those conducted scientifically (published literature) in order to elucidate how well this topic is being covered. We describe which species and behaviors the literature and social network inputs identify and emphasize areas for future research.

## 2. Methods

Social media observations were enumerated from 800 posts on the March for Science Facebook discussion and those from scientific literature came from 26 published studies which utilized observational protocols [37]. The published research was procured using the search terms “animal behavior during a solar eclipse” in Google Scholar, and by examining the literature cited in the studies identified by the Google Scholar search. The search results were screened to ensure a response was identified. Studies which concluded no behavioral response to the solar eclipse were omitted because it was presumed that the general public was less likely to report responses when none could be identified. Published studies of this type tended to be relatively brief and sample size was limited to those accessible through our university server and published in English.

In order to compare animal behavior observations obtained through diverse methods and a variety of sources, including social media [37] and published research, we independently classified responses into broader categories to facilitate comparison. We labeled a vocalization as any behavior associated with sound or an activity if it referred to movement. If behavior was described as commencing with the onset of an eclipse it was categorized as an increase and those associated with ceasing an activity during the eclipse were called decreases. This resulted in a total of four behavior classifications: vocalization increase, vocalization decrease, activity increase, and activity decrease. Animals noted in observations as “heading to the roost” or similar remarks were classified as decreases in activity since the overall reaction was to lessen their movement. We used these simplistic characterizations to create a common means of comparison across multiple diverse species. All characterizations were coded by two individuals—one coding the social media observations and the other the published literature—to maintain independence between the data sets. To reduce the potential for errors and coder bias, the two consulted with each other frequently to ensure they were consistent in how they coded different observations. While more sophisticated behavioral classifications would be advantageous in future investigations, we believed general characterizations were more appropriate given the diversity of species included in these observations and the potential unreliability of social media data.

Instances of a behavioral occurrence were enumerated by species and behavior classification with no regard for the number of individuals, as each report was counted as a single observation. However, if individuals in a group displayed different or multiple behaviors, each behavior was counted as a separate record. For example, if frogs were reported to both increase vocalization and activity, the record was counted twice: once for the increase in vocalization and once for the increase in activity. The finest taxonomic classification reported for an animal exhibiting a behavior was used as the animal name, but many online observations simplistically reported broader names such as “birds” or “frogs.” The differing scales of species classification alter the overall count of species behaviors, however assessing observations by animal group alleviates this issue. Additionally, given that we have no knowledge of the scientific knowledge of the social media participants, we did not feel we could fully trust their species-level identification. We classified animals in each observation as amphibian, bird, fish, invertebrate, mammal, or reptile. This eliminates inconsistency in naming, accounts for the potential unreliability of social media data, and allows for broad comparisons of observations between sources in addition to maintaining taxonomic consistency with broader analyses of animal behavior [1].

## 3. Results

### 3.1. Social Media: Anecdotal Observations of Behavior during the 2017 Eclipse

**Figure 1:**
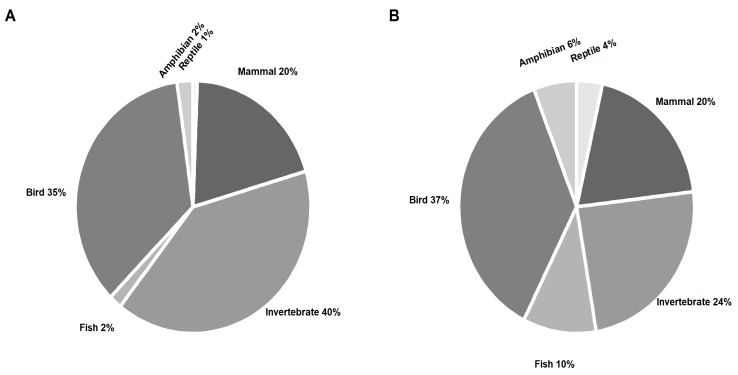
Proportions of observations made for different types of animals and behaviors recorded by social media and scientific literature. The proportion of animal groups observed recorded by social media during the 2017 Great American solar eclipse (**A**) and the scientific literature during multiple previous eclipses (**B**) is reported as a percent of the total number of observations made for each source (social media and scientific literature each total 100%). All observations were classified into one of six types of animals (amphibian, bird, fish, invertebrate, mammal, or reptile).

**Figure 2:**
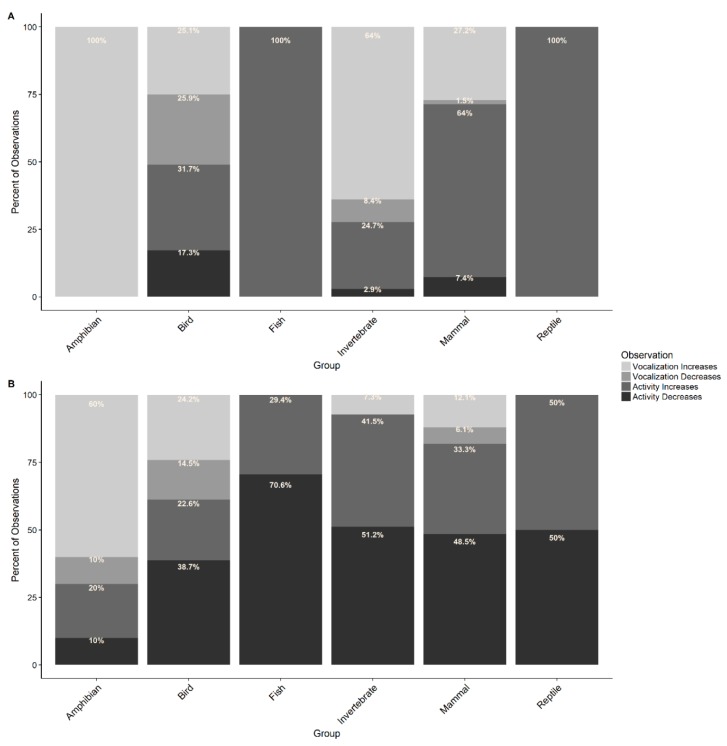
The proportion of behaviors observed for each group of animals recorded by social media (**A**) and scientific literature (**B**) is reported as a percent of the total number of observations made for each group of animals by the source (each animal grouping totals 100% in each source type). All observations were classified into one of four behaviors (vocalization increase, vocalization decrease, activity increase, or activity decrease).

A total of 685 observations of approximately 48 different types of animals reacting to the 2017 Great American Solar Eclipse were enumerated the March for Science Facebook discussion (Table 1) [37]. The animals most frequently reported on social media reacting to the eclipse were invertebrates (40% of social media observations) and birds (35% of social media observations; Figure 1A). The invertebrates included 11 types of insect as well as the broader category of ‘nocturnal insects’, spiders, and slugs. Crickets and cicadas received the highest proportion of reported observations at 33.8% and 31.6% of invertebrate observations, respectively. The most frequently reported behavior for invertebrates was vocalization increasing (64% of invertebrate observations) followed by decreases in activity (24.7% of invertebrate observations; Figure 2A). Observations of birds included 15 types in addition to the broader categories of “birds” and “seabirds”. The most commonly mentioned bird was chickens (15.6% of bird observations). The behavior most frequently reported was activity increasing (31.7% of bird observations) followed by decrease in vocalization (25.9% of bird observations; Figure 2A). Observations of mammals comprised 20% of the social media posts (Figure 1A), with the most commonly mentioned of the 15 types of mammals observed being dogs (24.3% of mammal observations) and bats (16.9% of mammal observations). An increase in activity was most frequently reported for mammals (64% of mammal observations) followed by increases in vocalization (27.2% of mammal observations; Figure 2A). Social media posts related to amphibians (2% of social media observations; Figure 1A) were comprised completely of reports of “frogs” which increased vocalization (Figure 2A) while observations of fish and reptiles (2% and 1% of social media observations, respectively; Figure 1A) only reported “fish” and “snakes” which increased activity (Figure 2A).

### 3.2. Scientific Literature: Published Observations of Behavior from Previous Eclipses

A total of 169 behavior observations for approximately 131 different animal species were recorded in the published literature (Table 2). The majority of observations came from Wheeler et al. (1935) [7] (54% of literature observations), Murdin (2001) (10.7% of literature observations) [38], and Kullenberg (1955) (10.6% of literature observations) [39], which also contained notes on a wide diversity of taxa, many of which were collected from the general public observations as well [7]. The remaining studies tended to have a narrower taxonomic focus and explicit hypotheses.

The group with the highest number of observations in the literature were birds with 62 records (37% of literature observations; Figure 1B) of 51 species responding to solar eclipse, the majority of which were chickens (7.8% of bird observations). Most observations reported decreases in activity (38.7% of bird observations) followed by increases in vocalization (24.2% of bird observations; Figure 2B). There were approximately 30 different species of invertebrate observed (24% of literature observations; Figure 1B), most frequently reported of which were zooplankton (14.6% of invertebrate observations). The most commonly reported behavior was a decrease in activity (51.2% of invertebrate observations) followed by activity increase (41.5% of invertebrate observations; Figure 2B). Interestingly, increases in vocalization were reported in 7.3% of observations while none mentioned decreases in vocalization (Figure 2B). The literature reported observations for approximately 22 different mammal species (20% of literature observations; Figure 1B) with the most numerous records being of dogs and gray squirrels (12.1% of mammal observations each) followed by cats (9.1% of mammal observations). The most commonly reported mammal behavior was a decrease in activity (48.5% of mammal observations) followed by increase in activity (33.3% of mammal observations; Figure 2B). There were 14 different species of fish recorded in the literature to respond to a solar eclipse (10% of literature observations; Figure 1B). The most common species to respond were the small-mouth black bass, goldfish, and brook trout (11.8% of fish observations each). The most frequently recorded behavior for fish was a decrease in activity (70.6% of fish observations; Figure 2B). Observations of amphibian species were least precisely reported but comprised of frogs and toads of various species and totaled ten observations in all (6% of literature observations; Figure 1B). The most frequently reported behavior change was an increase in vocalization (60% of amphibian observations; Figure 2B). Observations of reptiles comprised two species of lizard, three species of snake, and one species of turtle (4% of literature observations; Figure 1B). Each species had only one observation and reports of activity increase and decrease were recorded with equal frequency (50% of reptile observations each; Figure 2B).

## 4. Discussion

The scientific literature encompassed multiple eclipse events but contained fewer total observations than social media which only pertained to a single eclipse, however, the overall proportion of observations was similar for each animal group. In general, social media observations focused on increases in vocalization and activity while scientific literature favored activity over vocalization observations. By taxa, observations of birds, invertebrates, and mammals were more common than those of amphibians, fish, and reptiles. However, the literature tended to report a higher diversity of behavioral changes than social media.

There are numerous factors associated with a solar eclipse that may be the root cause of the observed behavioral changes. Not only is there a change in light, but also changes in air temperature and wind speed [4]. It is possible that the brief changes in temperature and wind speed are sensed by animals and, in combination with reduced light, are interpreted as the beginning of nocturnal changes or large storm, potentially enhancing bird and invertebrate vocalizations as instinctive behavior. The specific response by an individual of any species will depend on their specific life history and behavioral patterns associated with nocturnal changes [7] or a large storm, which can also decrease light via cloud cover.

Social media has potential utility to this kind of research because the large numbers observations gathered by this outlet can capture a wider spectrum of species and behaviors, highlighting topics of further exploration by researchers. The high prevalence of records for invertebrates and birds on social media is compelling for future research. Though covered in similar proportions within the literature relative to the other categories, additional scientific studies focusing on these taxa are likely to be fruitful. Considering the documented role celestial cues have on the orientation of birds [43,44,45] and insects [1,14], there are a number of potential factors to examine why these taxa would respond to such phenomena, which may also include behavioral responses to anomalies such as geomagnetic–electromagnetic changes (i.e., associated to impending earthquakes, i.e., [46]. It is also interesting to note that reports of decreases in invertebrate vocalization were found in social media but no such observations were present in the scientific literature. This may suggest an additional area of research requiring attention. Only a single type of behavior was reported for amphibians, fish, and reptiles through social media, which could be factor of people not looking at these species during an eclipse, regardless multiple responses were recorded in the scientific literature. It is worth noting for these taxa that even though the overall proportion of observations from these sources differed, they each had a similar total number of reports for these groups. Interestingly, a higher proportion of fish observations in the scientific literature reported a decrease in activity while only an increase in activity was reported on social media.

The major differences in behavioral observations between these sources may be due to the amateur nature of the social media records. Using social media, we do not know the participants or how reliable the information they provided may or may not be. This is a shortcoming of social media data, but given our goal of identifying gaps in the published research with regards to the taxonomic group or generalized behaviors that are potentially understudied or undervalued with regards to their response to a solar eclipse, we believe that the social media data was suitable. However, for more detailed analyses of specific species or behaviors, a more formalized citizen science approach would be necessary.

Though similar such accounts appear in the literature [7], the observational methodology for this type of research is congruent to how the social media investigations were conducted. The underlying mechanisms responsible for observed differences within and between groups is likely related to their specific life histories and characteristics. For instance, fish and reptiles do not typically produce vocalizations audible to casual observers, which supports why this response was not recorded in the literature (Table 2) or on social media (Table 1). Other influences of behavioral responses are extensively covered in the literature [1]. In order to test these differences and examine their causes, research on this question needs to become more rigorous. This requires a shift from observational accounts, which dominate the literature, to experimental, quantitative investigations of why, how, and when animals respond to solar eclipses or other natural phenomena. It is clear that more research is necessary to disentangle the numerous influences responsible for these observed behaviors. Such investigations can contribute to the advancement of animal behavior science by increasing our understanding of how external factors influence activity.

### Recommendations for Future Research

It is evident from both the literature and anecdotal observations that behavioral responses of many animals to solar eclipses are highly dependent on the specific animal in question. Overall, our findings indicate that activity generally changes more frequently than vocalization, except for amphibians. Organisms which seem to be understudied in relation to this phenomenon are amphibians, fish, and reptiles. Though all taxa in question need to be further explored to elucidate causes of their responses, these particular groups lack the most in behavioral studies during an eclipse. However, birds and invertebrates seem to offer the richest prospects for these investigations due to their prevalence in accounts from both sources. In order to make reasonable conclusions about an animal’s behavioral response to a solar eclipse, a detailed understanding of its life history and prevailing environmental conditions during the period are necessary [7]. Whether an animal responds may also be related to the sensitivity of its underlying biological rhythms to environmental factors [1]. Researchers need to compose clear hypotheses concerning expected and observed behavioral responses based on their specific study organism in order to put their responses into context. It is helpful to consider this type of research as special cases of biological rhythms [15], which can serve as basis for designing experiments to test behavioral responses to solar eclipses. These future experiments should seek to implement new methods and technologies for recording animal behavior, such as GPS transmitters, thermal cameras, drones, etc. Our understanding of animal behavior can progress beyond the narrow scope of current studies by characterizing the complex variations in behavioral response which result from a solar eclipse.

## Figures and Tables

**Table 1:** Total number of animal behavior observations enumerated from March for Science Facebook discussion during 2017 Great American Solar Eclipse.

Animal	Group	Vocalization Increase	Vocalization Decrease	Activity Increase	Activity Decrease
Frogs	Amphibian	15	0	0	0
Birds	Bird	27	53	14	26
Chicken	Bird	13	9	7	9
Cranes	Bird	1	0	1	0
Crows	Bird	7	0	7	0
Geese	Bird	2	0	3	3
Hawk	Bird	2	0	5	1
Humming birds	Bird	0	0	9	0
Martin	Bird	0	0	2	0
Nighthawk	Bird	0	0	6	0
Owl	Bird	8	0	0	0
Pelicans	Bird	0	0	2	0
Seabirds	Bird	0	0	4	1
Seagulls	Bird	1	1	3	0
Starling	Bird	0	0	4	0
Swallow	Bird	0	0	6	1
Turkey	Bird	0	0	3	0
Vultures	Bird	0	0	1	1
Fish	Fish	0	0	12	0
Ants	Invertebrate	0	0	3	0
Bees	Invertebrate	0	2	18	5
Cicadas	Invertebrate	73	14	0	0
Crickets	Invertebrate	86	7	0	0
Dragon flies	Invertebrate	0	0	7	0
Fireflies	Invertebrate	0	0	10	0
Gnats	Invertebrate	0	0	8	1
Grasshopper	Invertebrate	0	0	0	1
Locust	Invertebrate	2	0	0	0
Mosquitos	Invertebrate	0	0	16	0
Nocturnal insects	Invertebrate	15	0	2	0
Slug	Invertebrate	0	0	1	0
Spiders	Invertebrate	0	0	3	1
Bat	Mammal	0	0	23	0
Cat	Mammal	0	0	8	0
Cows	Mammal	5	0	3	2
Coyote	Mammal	14	0	0	0
Deer	Mammal	0	0	7	1
Dogs	Mammal	11	0	22	0
Dolphins	Mammal	0	0	2	0
Elk	Mammal	0	0	1	0
Giraffes	Mammal	0	0	2	0
Horses	Mammal	2	0	10	2
Prairie Dog	Mammal	1	0	1	0
Rabbits	Mammal	0	0	5	0
Sheep	Mammal	0	1	1	0
Squirrels	Mammal	0	1	2	5
Wolves	Mammal	4	0	0	0
Snakes	Reptile	0	0	4	0

**Table 2:** Total number of observations reporting changes in animal behavior during eclipses enumerated from published research.

Animal	Group	Citation	Vocalization Increase	Vocalization Decrease	Activity Increase	Activity Decrease
American Toad	Amphibian	[7]	0	0	1	1
Bullfrog	Amphibian	[7]	0	0	1	0
Frog	Amphibian	[7,38]	2	1	0	0
Tree frog	Amphibian	[40]	1	0	0	0
Tree toad	Amphibian	[7]	1	0	0	0
Troschel’s tree frog	Amphibian	[41]	1	0	0	0
Barred owl	Bird	[7]	1	0	0	0
Black-Crowned Night Heron	Bird	[7]	0	0	1	0
Blue jay	Bird	[7]	0	1	0	0
Bronzed grackle	Bird	[7]	0	0	0	1
Brown pelican	Bird	[9]	0	0	0	1
Bulbuls	Bird	[38]	0	1	0	0
Cattle Egrets	Bird	[8]	0	0	1	0
Chicken	Bird	[7,40]	2	0	1	1
Common blackbird	Bird	[39]	1	0	0	0
Common chaffinch	Bird	[39]	0	1	0	0
Common tern	Bird	[7]	1	0	0	0
Crow	Bird	[7,40]	0	0	1	2
Dowitcher	Bird	[7]	1	1	1	0
Ducks	Bird	[7]	0	0	1	0
Egret	Bird	[38]	0	0	0	1
Eurasian blackcap	Bird	[39]	1	0	0	0
European robin	Bird	[39]	1	0	1	0
Fish hawk	Bird	[7]	1	0	1	0
Garden warbler	Bird	[39]	0	1	0	0
Geese	Bird	[38]	0	0	0	1
Glossy starlings	Bird	[38]	0	1	0	0
Goldfinch	Bird	[7]	0	0	0	1
Great Egrets	Bird	[8]	0	0	1	0
Herring gull	Bird	[7]	0	0	0	1
Ibis	Bird	[38]	0	0	0	1
Least sandpiper	Bird	[7]	0	0	0	0
Little Blue Herons	Bird	[8]	0	0	1	0
Magnificent frigate-bird	Bird	[9]	0	0	0	1
Northern flicker	Bird	[7]	0	0	0	1
Owl	Bird	[38]	1	0	0	0
Oxpecker	Bird	[38]	0	0	0	1
Pectoral sandpiper	Bird	[7]	0	0	0	1
Red-winged blackbird	Bird	[7]	0	0	0	1
Robin	Bird	[7]	0	0	0	1
Roseate tern	Bird	[7]	1	0	0	0
Royal tern	Bird	[9]	0	0	0	1
Screech owl	Bird	[7]	1	0	0	0
Semipalmated plover	Bird	[7]	0	0	0	1
Semipalmated sandpiper	Bird	[7]	0	0	0	1
Snowy egrets	Bird	[8]	0	0	1	0
Song thrush	Bird	[39]	0	1	0	0
Starling	Bird	[7]	0	0	1	0
Trumpeter hornbill	Bird	[38]	0	0	0	1
Turtle-dove	Bird	[38]	0	1	0	0
Water birds	Bird	[38]	0	0	0	1
Whip-poor-will	Bird	[7]	1	0	0	0
Willet	Bird	[7]	1	0	1	0
Willow warbler	Bird	[39]	0	1	0	0
Wilson’s petrel	Bird	[7]	0	0	0	1
Angelfish	Fish	[25]	0	0	0	1
Anthias	Fish	[25]	0	0	0	1
Banded gourami	Fish	[24]	0	0	0	1
Brook Trout	Fish	[7]	0	0	1	1
Butterflyfish	Fish	[25]	0	0	0	1
Climbing perch	Fish	[24]	0	0	0	1
Common Pickerel	Fish	[7]	0	0	1	0
Damselfish	Fish	[25]	0	0	0	1
Goldfish	Fish	[7]	0	0	1	1
Hawkfish	Fish	[25]	0	0	0	1
Mud eel	Fish	[24]	0	0	0	1
White perch	Fish	[7]	0	0	1	0
Wrasse	Fish	[25]	0	0	0	1
Bees	Invertebrate	[38]	0	0	0	1
Bumblebees	Invertebrate	[7]	0	0	0	1
Butterflies	Invertebrate	[7,38]	0	0	0	2
Cercariae	Invertebrate	[42]	0	0	1	0
Cicadas	Invertebrate	[7]	1	0	0	0
Cockroaches	Invertebrate	[7]	0	0	1	0
Crabro vagus	Invertebrate	[39]	0	0	0	1
Crickets	Invertebrate	[7]	1	0	0	0
Dinoflagelletes	Invertebrate	[15]	0	0	1	0
European rose chafer	Invertebrate	[39]	0	0	0	1
Fucellina fly	Invertebrate	[40]	0	0	0	1
Glanville fritillary	Invertebrate	[39]	0	0	0	1
Gnats	Invertebrate	[7]	0	0	1	0
*Gorytes campestris*	Invertebrate	[39]	0	0	0	1
*Gorytes mystaceus*	Invertebrate	[39]	0	0	0	1
Grasshoppers	Invertebrate	[7,39]	0	0	0	2
*Hoplomerus melanocephalus*	Invertebrate	[39]	0	0	0	1
Houseflies	Invertebrate	[7]	0	0	1	1
Katydids	Invertebrate	[7]	1	0	0	0
Large-spurred digger wasp	Invertebrate	[39]	0	0	0	1
Midge	Invertebrate	[38,40]	0	0	2	0
Mosquitoes	Invertebrate	[7,38]	0	0	2	0
Moths	Invertebrate	[7]	0	0	1	0
Orb-weaving spider	Invertebrate	[13]	0	0	1	0
Rock bees	Invertebrate	[10]	0	0	1	0
Sahara Desert ant	Invertebrate	[11]	0	0	0	1
Slender bodied digger wasp	Invertebrate	[39]	0	0	0	1
Wasps	Invertebrate	[7]	0	0	0	1
Zooplankton	Invertebrate	[16,17,18,19]	0	0	4	2
Antelope ground squirrel	Mammal	[26]	0	0	1	0
Baboon	Mammal	[38]	0	0	0	1
Bat	Mammal	[7]	0	0	1	0
Beaver	Mammal	[7]	0	0	1	1
Blue Bull Antelope	Mammal	[32]	0	0	0	1
Bush rat	Mammal	[27]	0	0	0	1
Chimpanzee	Mammal	[20]	0	0	1	0
Guinea-pig	Mammal	[7]	0	0	0	1
Hamadryas baboon	Mammal	[21]	0	0	0	1
Hippopotamus	Mammal	[38]	0	0	1	0
Impala	Mammal	[38]	0	0	0	1
Proboscis Monkey	Mammal	[23]	0	0	0	1
Red fox	Mammal	[7]	1	0	1	0
Rhesus monkey	Mammal	[7]	0	0	0	1
Skunk	Mammal	[7]	0	0	1	0
Eastern garter snake	Reptile	[7]	0	0	1	0
North American side-blotched lizard	Reptile	[31]	0	0	1	0
Northern watersnake	Reptile	[7]	0	0	0	1
Painted turtle	Reptile	[7]	0	0	0	1
Pythons	Reptile	[7]	0	0	1	0
Zebra-tailed lizard	Reptile	[30]	0	0	0	1
